# Editorial: Understanding the role of microbiome in alteration of cellular metabolism and cancer development

**DOI:** 10.3389/fcimb.2025.1686038

**Published:** 2025-09-30

**Authors:** Lovlesh Thakur, Sunil Thakur, Vibha Rani, Shiv Verma, Prem Prakash Kushwaha

**Affiliations:** ^1^ Department of Biological, Geological, and Environmental Sciences, Cleveland State University, Cleveland, OH, United States; ^2^ Origin LIFE Healthcare Solutions and Research Center, Chandigarh, India; ^3^ Center for Emerging Diseases, Department of Biotechnology, Jaypee Institute of Information Technology, Uttar Pradesh, India; ^4^ Department of Urology, Case Western Reserve University, Cleveland, OH, United States

**Keywords:** microbiome-cancer interactions, metabolic dysregulation, microbial dysbiosis, multi-omics analysis, precision oncology

## Introduction

Cancer is not just a genetic disease but is deeply entangled with trillions of microbes that live in and on our bodies. The human microbiome has emerged as a critical regulator of cellular metabolism and, increasingly, as a driver of carcinogenesis across multiple organ systems. In this editorial, we provide a concise overview of the intricate relationship between the microbiome and carcinogenesis, underscoring several unifying insights crucial for understanding this emerging field. The ten papers published in frontiers research editorial topic titled “Understanding the Role of Microbiome in Alteration of Cellular Metabolism and Cancer Development,” explored the surprising connection between human microbiome and cancer-ranging from narrative and systematic reviews to original mendelian randomization and multi-omics studies. These studies guide us, how microbial communities and their metabolites modulate tumor initiation, progression, and treatment response. Here, we have summarized each contribution and suggested future directions for this rapidly evolving field.

Sabour et al. comprehensively reviewed, how anaerobic bacteria including *Fusobacterium nucleatum*, *Enterotoxigenic Bacteroides fragilis*, *Peptostreptococcus, Prevotella*, *Clostridium* spp., and others initiate and exacerbate colorectal cancer (CRC). Under low-oxygen conditions of a tumor, these bacteria release toxins and metabolites that activate multiple mechanisms by which anaerobic bacteria drive carcinogenesis: activation of Wnt/β-catenin signaling, disruption of epithelial architecture, proinflammatory cytokine induction, and direct genotoxic DNA damage in hypoxic tumor niches, essentially providing a local environment to favor tumor growth. The authors underscore the role of dysbiosis and aberrant microbial metabolites (e.g., secondary bile acids, short-chain fatty acids) in shaping a pro-tumorigenic microenvironment.

Chen et al. provided a comprehensive review that links the gut and intratumorally microbiota dysbiosis and gastric cancer (GC). They have highlighted the pivotal role of microbial dysbiosis in the initiation and progression of GC, wherein; imbalance in the microbial community notably *Helicobacter pylori* alongside enrichment of *Citrobacter*, *Clostridium*, *Fusobacterium*, and others contribute to GC by promoting genomic instability, impaired DNA repair, exacerbating tumor hypoxia, and fostering an immunosuppressive microenvironment. The authors evaluate the translational aspect of potential microbiota-based interventions including probiotics, prebiotics, antibiotics, fecal transplantation, and traditional Chinese medicine to improve the chemotherapeutic and immunotherapeutic outcomes. The authors also highlighted the need for rigorous preclinical and clinical validation of these strategies.

Jin and Zhong integrated the transcriptomic profiles with gut microbiome data to colorectal cancer. Based on their analysis they classified colorectal cancer into two biologically and clinically distinct tumor subtypes and constructed a risk score model. The one group, marked by favorable prognosis, in contrasts to another group, which harbors a richer gut microbiota and poorer outcomes. The authors risk-scoring model effectively stratifies survival and links microbial composition to immune microenvironment features and therapeutic sensitivity. Distinct taxa including *Robiginitomaculum* and *Myxococcus* in the low-risk group, and *Sutterella* and *Zymomonas* in the high-risk group emerge as potential biomarkers. This work represents a meaningful step toward the critical importance of personalized treatment.

A mendelian randomization study investigates whether gut microbes can causally influence the risk of ovarian cancer through metabolic changes. Using large‐scale Genome-Wide Association Study (GWAS) datasets for gut microbiota, blood metabolites, and ovarian cancer, the authors identify turicibacter sp001543345 as a protective player. Its presence appears to affect lipid metabolism by (1) lowering free cholesterol in small HDL and raising saturated fatty acid ratios to total fatty acids, (2) increasing total cholesterol and cholesteryl esters to total lipids ratio in very‐small VLDL particles changes that collectively reduce cancer risk. Additionally, they mapped the genetic-microbial-metabolic chain that pinpoints lipoprotein subclasses and fatty acid ratios as key mediators. Such specificity suggests a future where microbial profiling and targeted lipid modulation could form part of ovarian cancer prevention strategies. By bridging genetics, microbiology, and lipid biochemistry, the study offers a compelling glimpse of precision cancer prevention grounded in the microbiome (Zhang et al.).

Gu et al. use mendelian randomization to show a causal link between specific oral bacteria and CRC risk using data from two large east Asian databanks including China national gene bank and biobank Japan. Using multi-omics approach, they identify 19 taxa that exhibit strong causal associations, with some such as RUG343 appearing protective and others like HOT-345_umgs_976 and W5053_sp000467935_mgs_712 increasing susceptibility. Single-cell RNA sequencing reveals that high-risk microbial profiles are associated with activation of JAK-STAT signaling and tyrosine metabolism in tumor-adjacent endothelial cells. These findings suggest that oral microbes may influence CRC development through metabolic and inflammatory pathways. They performed *in silico* drug screening and predicted potential therapeutic agents, including menadione sodium bisulfite and raloxifene, that could target these mechanisms and interrupt the microbe driven pathways. This integrated genetic, cellular, and drug-prediction approach opens new possibilities for CRC prevention and treatment.

In a study, Han and Fan unfold the complexity of how shifts in vaginal microecology align with human papillomavirus (HPV) infection and cervical lesion progression. Using Illumina high-throughput sequencing targeting the V4 region of the 16S rRNA gene, they characterized microbial communities across healthy women, high-risk HPV carriers, and those with cervical intraepithelial neoplasia (CIN). Their data reveal that although overall species richness of vaginal microbes doesn’t change, cervical cancer (CC) patients exhibit significantly higher microbial diversity compared to high-risk HPV carriers or those with CIN. CC patients showed decline in *Lactobacillus* and Cyanobacteria and a rise in genera such as *Dialister* and *Peptoniphilus*. Crucially, the research identifies stage-specific biomarkers, including *Varibaculum* in healthy women, *Saccharopolyspora* in individuals with high-risk HPV, and several taxa such as *Coprococcus*, *Peptococcus*, and *Ruminococcus* in cervical cancer. These biomarkers suggest that vaginal microbial composition could serve as a diagnostic adjunct for distinguishing between infection stages and cervical lesions. The findings hint that restoring a *Lactobacillus*-dominated microbiome might not only help manage HPV infection but also slow or prevent progression to cervical cancer. This study stands out by providing a proposing tangible microbial target for early intervention of cancer.

Cao et al. presents a narrative review on evidence linking gut microbial alterations to benign prostatic hyperplasia and prostate cancer. The authors synthesize mounting observational and animal data linking gut dysbiosis to chronic prostatitis, benign prostatic hyperplasia, and prostate cancer. Dysbiosis induced changes in circulating short-chain fatty acids (SCFAs), androgen metabolism, and systemic inflammation are proposed as drivers of prostate cell proliferation and malignant transformation. The review highlights emerging data on fecal metabolite signatures as noninvasive biomarkers and therapeutic frontier in men’s urological health. Lastly, the review advocates for controlled trials of microbiota-modulating agents in prostatic disease.

Biliary atresia, devastating cholangiopathy remains one of the most challenging pediatric liver diseases, with a complex interplay of genetic, immune, infectious, and environmental factors contributing to its onset and progression. Feng et al. reviewed the role of gut microbiota and their metabolites in biliary atresia in children. Recent research has brought the gut microbiome into sharper focus, revealing how microbial imbalance and altered metabolites may disrupt bile acid metabolism, trigger inflammation, and influence post-surgical outcomes. This review highlights how interventions targeting gut bacteria through probiotics, prebiotics, or microbial metabolites like butyrate could complement current surgical treatments such as Kasai hepatoportoenterostomy. While promising, these strategies require robust clinical validation to confirm their safety, efficacy, and long-term benefits. Understanding the gut-liver axis in biliary atresia could pave the way for more personalized, effective, and preventative approaches.

Yan et al. used 16S rRNA sequencing and LC-MS metabolomics to profile gut microbiota and serum metabolites in post-menopausal women with normal and reduced bone mineral density (BMD). They divided the individuals into normal and osteoporosis (OS) group. They found higher microbial richness in low-BMD women, with *Bacteroides*, *Blautia*, *Ruminococcus*, and *Anaerostipes* linked to higher BMD, while *Agathobacte*r and *Lactobacillus* were enriched in low-BMD cases. Metabolomics revealed alterations in tryptophan metabolism, fatty acid degradation, and steroid hormone biosynthesis. Notably, Bacteroides abundance correlated with the microbial tnaA gene, connecting gut metabolism to bone health. A combined microbial-metabolite model accurately predicted low-BMD status. These findings highlight multi-omics biomarkers and pathways that may guide microbiome-targeted strategies for osteoporosis prevention in postmenopausal women.

Liang et al. employed bidirectional mendelian randomization approach to test causal relations between gut microbiota changes and lymphoma risk. They used robust method utilizing large-scale GWAS data and identify specific bacterial taxa that appear to increase or reduce the risk of distinct lymphoma subtypes, from diffuse large B-cell lymphoma to Hodgkin lymphoma. microbes like *Alistipes*, *Turicibacter*, *Lactobacillus*, and *Akkermansia* showing protective effects, while *Ruminococcaceae UCG002*, *Eubacterium ventriosum group*, and *Phascolarctobacterium* increased risk. These effects may act through immune modulation, including regulation of immune cell balance, influencing inflammation and antitumor immunity. Microbial metabolites such as short-chain fatty acids particularly butyrate were implicated in key pathways like NF-κB inhibition, histone acetylation, and apoptosis induction. These findings pave the way for microbiome-based preventive strategies in hematologic malignancies.

As we look ahead, the convergence of high‐throughput multi‐omics, causal inference methods, and precision microbiology will be critical for harnessing the microbiome in cancer prevention, diagnosis, and therapy. The studies summarized here lay a robust foundation and chart a clear path for the next wave of microbiome‐centric oncology research.

